# Proposed Fatigue Index for the Objective Detection of Muscle Fatigue Using Surface Electromyography and a Double-Step Binary Classifier

**DOI:** 10.3390/s22051900

**Published:** 2022-02-28

**Authors:** Hassan M. Qassim, Wan Zuha Wan Hasan, Hafiz R. Ramli, Hazreen Haizi Harith, Liyana Najwa Inche Mat, Luthffi Idzhar Ismail

**Affiliations:** 1 Department of Electrical and Electronic Engineering, Faculty of Engineering, Universiti Putra Malaysia, Serdang 43400, Selangor, Malaysia; hassanmessar@gmail.com (H.M.Q.); hrhr@upm.edu.my (H.R.R.); luthffi.ismail@upm.edu.my (L.I.I.); 2 Department of Medical Instrumentation Engineering, Technical Engineering College of Mosul, Northern Technical University, Mosul 41001, Iraq; 3 Department of Biological and Agricultural Engineering, Faculty of Engineering, Universiti Putra Malaysia, Serdang 43400, Selangor, Malaysia; hazreen@upm.edu.my; 4 Department of Neurology, Faculty of Medicine and Health Sciences, Universiti Putra Malaysia, Serdang 43400, Selangor, Malaysia; liyananajwa@upm.edu.my

**Keywords:** electromyography, fatigue, fatigue index, muscle, non-fatigue

## Abstract

The objective detection of muscle fatigue reports the moment at which a muscle fails to sustain the required force. Such a detection prevents any further injury to the muscle following fatigue. However, the objective detection of muscle fatigue still requires further investigation. This paper presents an algorithm that employs a new fatigue index for the objective detection of muscle fatigue using a double-step binary classifier. The proposed algorithm involves analyzing the acquired sEMG signals in both the time and frequency domains in a double-step investigation. The first step involves calculating the value of the integrated EMG (IEMG) to determine the continuous contraction of the muscle being investigated. It was found that the IEMG value continued to increase with prolonged muscle contraction and progressive fatigue. The second step involves differentiating between the high-frequency components (HFC) and low-frequency components (LFC) of the EMG, and calculating the fatigue index. Basically, the segmented EMG signal was filtered by two band-pass filters separately to produce two sub-signals, namely, a high-frequency sub-signal (HFSS) and a low-frequency sub-signal (LFSS). Then, the instantaneous mean amplitude (IMA) was calculated for the two sub-signals. The proposed algorithm indicates that the IMA of the HFSS tends to decrease during muscle fatigue, while the IMA of the LFSS tends to increase. The fatigue index represents the difference between the IMA values of the LFSS and HFSS, respectively. Muscle fatigue was found to be present and was objectively detected when the value of the proposed fatigue index was equal to or greater than zero. The proposed algorithm was tested on 75 EMG signals that were extracted from 75 middle deltoid muscles. The results show that the proposed algorithm had an accuracy of 94.66% in distinguishing between conditions of muscle fatigue and non-fatigue.

## 1. Introduction

Fatigue is defined as the inability of the respective muscles to contract and perform a specific procedure over a long period of time [1,2]. This inability happens because of the reduction of the central motor drive of the muscle, which reduces the amount of force being produced, and consequently results in the experience of pain and fatigue [3].

Despite the fact that the assessment of muscle fatigue based on an analysis of the surface electromyography (sEMG) signal has been intensively discussed and understood [4], the objective detection of muscle fatigue still requires further investigation [5]. In medical terms, muscle fatigue is detected either subjectively or objectively [6]. The subjective detection of muscle fatigue is reported by the individuals themselves, when no type of measurement is applied [6]. However, the objective detection of muscle fatigue requires the application of a specific type of measurement to detect the fatigue, which, in the case of this study, involves an analysis of the sEMG signals [7]. Consequently, the objective detection of muscle fatigue disregards any subjective reports by individuals [7], and, furthermore, it identifies the level of exhaustion of the muscle [8]. Hence, the objective detection of muscle fatigue is able to prevent post-fatigue injuries caused by over-training by athletes and rehabilitation exercises for stroke patients [9,10].

In surface electromyography (sEMG), electrical signals from a muscle are captured by electrodes attached directly to the surface of the skin. The acquired sEMG signal must be accurately processed and analyzed to interpret the information contained within. The method of processing and analyzing is determined based on the dedicated purpose of the extracted sEMG signal [11]. Currently, EMG is analyzed either in a time domain or frequency domain. An analysis of the EMG in a time domain involves extracting several amplitude-time dependent features, such as the root mean square (RMS) [12], integrated EMG (IEMG) [13], absolute rectified value (ARV) [14], log detector (LOG) [15], waveform length (WL) [16], zero crossing (ZC) [17], slope sign change (SSC), etc. [18]. Time domain features are mostly employed to provide fine controls for prostheses and rehabilitation robots [19,20,21]. However, analyzing in a frequency domain involves extracting power-frequency dependent features, such as the mean frequency (MNF), median frequency (MDF) [18], total power (TTP) [13], etc. [16]. Frequency domain features are mostly dedicated to the assessment of muscle fatigue [4]. The analysis of the EMG in both domains for the purpose of controlling and assessing fatigue has been well studied, and several papers have been published on the subject [5,11,22].

For an EMG-based fatigue analysis, static and dynamic exercises are usually performed to activate the skeletal muscles of the body and force them to reach fatigue levels. For example, a dumbbell curl exercise is performed to activate the biceps and triceps [8]. Dumbbells of various weights, such as 5 kg, 6 kg, and 8 kg, are used to accelerate the progress of fatigue. It has been suggested that fatigue occurs once the subject can no longer perform the dumbbell curl activity [8,23]. Moreover, a hand muscle developer can be used to activate the forearm muscles [24], while the scapular plane is considered as an efficient posture for activating the middle deltoid muscle [25]. To conclude, whether static or dynamic exercises are performed, fatigue occurs once the muscle can no longer perform the activity. The next section reviews the literature and the related algorithms that were suggested for the objective detection of muscle fatigue.

### Literature Review and Motivation

Only a few types of research were suggested for the objective detection of muscle fatigue. Karthick and his colleagues [26] used the support vector machine classifier to classify fatigue and non-fatigue conditions, where an accuracy of 91% was achieved. The aforementioned study proposed the use of the extended modified B-distribution method of analysis to extract four features, namely, the singular value decomposition (SVD)-based entropy, kurtosis, mean frequency, and median frequency.

Fernando proposed an algorithm that combines two EMG features, namely, the mean frequency (MNF) and absolute rectified value (ARV), to objectively detect fatigue in the biceps muscle [23]. The MNF/ARV ratio is calculated and regarded as an index of muscle fatigue, where the aforementioned ratio gradually decreases as fatigue progresses [23]. Fatigue exists once the ratio reaches a specific baseline, which is defined as the inverse value of the MNF/ARV. As reported by the authors, the initial values of the MNF and ARV are taken as the reference values for the baseline to eliminate individual differences. Kiryu and his colleagues also used the MNF and ARV features to objectively detect fatigue [27]. The correlation coefficient between MNF and ARV is taken as an index of fatigue. The conversion of the correlation coefficient from positive to negative is regarded as a sign of muscle fatigue. The aforementioned algorithms have their own drawbacks that are either related to the computation time or to individual differences.

The mean power frequency (MNP) was proposed as a muscle fatigue index by [2,8]. The study suggested that a high-frequency spectrum tends to transform to a low-frequency spectrum with the progression of fatigue. The opposite behavior takes place when the muscle recovers, where the low-frequency spectrum tends to transform to a high-frequency spectrum. This study, however, only showed how to assess fatigue, which is widely identified by further frequency domain features, such as the MNF and MDF [16].

However, refs. [28,29] followed a different technique to detect muscle fatigue. They suggested that the frequency spectrum of the EMG can be classified into three regions, namely, low-frequency components (LFC) (20–45 Hz), intermediate-frequency components (IFC) (46–80 Hz), and high-frequency components (HFC) (81–350 Hz). The power spectrum of each component is calculated and their behavior is monitored. It was suggested that during fatigue, the power of the LFC increases, while that of the HFC decreases [30]. Fatigue occurs when the power of the LFC is equal to the power of the HFC [23]. However, such a classification of EMG frequency bands has its own drawbacks. For instance, the LFC could have a higher power than the HFC without there being any progress in fatigue. Furthermore, fatigued and non-fatigued muscles cannot be differentiated based on the aforementioned study [23].

In terms of classifying the EMG frequency bands, refs. [31,32] suggested that the frequency spectrum of the EMG can be classified into two regions, namely, low-frequency components (LFC) (20–46.7 Hz) and high-frequency components (HFC) (150–350 Hz), where the amplitude of the LFC tends to increase during fatigue, while the amplitude of the HFC tends to decrease. Similarly, ref. [13] further classified the frequency spectrum into four regions that lie within the following bands, namely, 24–56 Hz, 63–96 Hz, 104–136 Hz, and 144–800 Hz. However, an accurate identification of the muscle fatigue index was not explained in the aforementioned studies.

The rest of the paper is arranged as follows: Section 2 explains the materials and method in detail, including the proposed fatigue index; Section 3 discusses the results; while Section 4 concludes the study.

## 2. Materials and Method

This section explains the identification of the sample size, the appropriate muscle that was subjected to fatigue, and how fatigue was induced. Furthermore, the experimental setup, signal processing, the proposed classifier and the fatigue index will also be clarified. The following subsections explain the study in detail.

### 2.1. Sample Size

Identifying the optimum sample size is considered as an important step in testing the efficiency of any proposed algorithm. Ref. [33] Proposed an equation to calculate the required sample size. This equation is mathematically represented as follows:

(1) $$N=\frac{4\;{\left(Z_{crit}\right)}^{2}\rho \left(1-\rho \right)}{D^{2}}$$

where N is the number of required samples (sample size), and *Zcrit* is the significance criterion representing the value at which the difference is considered to be statistically significant. The *Zcrit* was identified by [33] and set to a value of 1.96. Finally, ρ is the proportional estimate of the preliminary studies. Therefore, the mean accuracy of the preliminary studies was first calculated (Table 1).

Considering that 95% was the chosen confidence interval with an interval of ±10%, hence *D* was equal to 0.2. Consequently, the sample size was calculated as follows:

$$N=\frac{4\;{\left(1.96\right)}^{2}\;0.89\left(1-0.89\right)}{0.2^{2}}$$

N = 37.6 ≈ 38 (number of required samples).

### 2.2. Choosing the Appropriate Muscle and Fatigue Classification

The deltoid muscles are the large muscles covering the shoulder, and their functions are to abduct, adduct, flex, and extend the upper extremities [25,40]. The deltoid muscles consist of three main muscles, namely, the anterior deltoid, middle deltoid, and posterior deltoid [40]. However, the middle deltoid muscle contributes to most of the movements in the upper limbs, and is responsible for elevating the upper limbs, which makes it susceptible to rapid fatigue [41]. Therefore, the middle deltoid muscle was chosen to evaluate the proposed algorithm for the objective detection of muscle fatigue. Localized muscle fatigue has been classified into four grades of fatigues [42] as follows:

I. Slight muscle cramp or tightening.
II. Sustained muscle cramp with a sort of painful feeling.
III. A continuous feeling of burning pain.
IV. Further painful feeling and lack of ability to maintain the activity.

Hence, a specific protocol was proposed to ensure that the middle deltoid muscle reached fatigue level, where the subject could no longer maintain the protocol. It should be noted that muscle pain and localized muscle fatigue are all alternatives to muscle fatigue [32].

### 2.3. Preparation of Subjects and Protocol Performance

As explained in the section on sample size, 38 subjects were selected for participation in this study. Their right and left middle deltoid muscles were tested with the proposed algorithm. The mean age of the participants was 34.2 ± 8.5 years. At the start of the study, the participants reported no injuries or pain in their middle deltoid muscle. However, the third subject reported moderate pain in her right middle deltoid, and, hence, was excluded from the experiment. Ultimately, 75 sEMG samples extracted from 75 middle deltoid muscles were included in this study.

**Figure 1:**
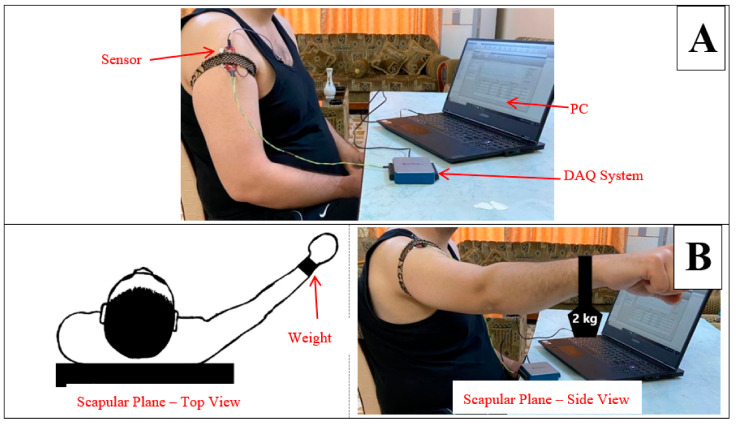
Protocol for performing the two muscle states: (**A**) non-fatigued muscle, and (**B**) fatigued muscle.

The experimental protocol involved requesting all subjects to sit on a chair and perform two types of movements in relation to the middle deltoid muscle as follows:

I. Muscle in Non-fatigue Condition: The subjects were asked to relax their middle deltoid muscles by performing no action (Figure 1A) and, simultaneously, the sEMG signal was recorded. This procedure was aimed at evaluating the performance of the proposed algorithm in detecting the non-fatigued muscle. Despite that the subjects performed no action in this posture, the middle deltoid muscle still played an important role in stabilizing the shoulder joint [40,43]. Eventually, the middle deltoid was not fully relaxed and exhibited a sort of sEMG signal.
II. Muscle in Fatigue Condition: The subjects were asked to elevate their upper limbs and keep them in the scapula plane with a 2 kg weight clutched on their forearms (Figure 1B). The subjects were encouraged to perform this activity until they could no longer maintain this posture, whereby the muscle was considered to have reached fatigue level. It should be noted that the weight was added to accelerate the generation of fatigue. This procedure was aimed at evaluating the performance of the proposed algorithm with regard to the objective detection of muscle fatigue.

The subjects were allowed to rest for five minutes in between sessions to relax their middle deltoid muscles [25,44]. Moreover, the scapula plane was chosen because of its efficiency in activating the middle deltoid muscle [25,42]. The aforementioned protocol was approved by the Ethics Committee of Universiti Putra Malaysia, Selangor, Malaysia with reference number (JKEUPM-2021-263).

### 2.4. Acquisition of the sEMG and Hardware Setup

A Myoware muscle sensor was placed on the middle deltoid muscle and used to collect the sEMG signals. The Myoware sensor was previously investigated and displayed its ability to convert the electrical activity in the muscle to an analogue signal [45]. Table 2 summarizes the parameters of the chosen sensor.

This chosen sensor is characterized by its three bio-potential pins, two of which are differential pins and one a reference pin. The three pins were connected to Covidien disposable electrodes (H124SG) for better acquisition of the sEMG signals [46]. As shown in Figure 1, the two differential electrodes of the sensor were attached in a straight line over the belly of the middle deltoid [47,48]. However, the reference electrode was attached to a different tissue, which was the anterior deltoid muscle in the case of this study [45]. Furthermore, the skin was first shaved and cleaned with alcohol prior to the application of the Covidien electrodes [49], while a tie was used to ensure that the electrode attachments were fixed on the muscle [24]. The guidelines of the International Society of Electrophysiology and Kinesiology (ISEK) [50] and those of the European Recommendations for Surface Electromyography (SENIAM) [51] were followed for better acquisition of the sEMG signals.

**Figure 2:**
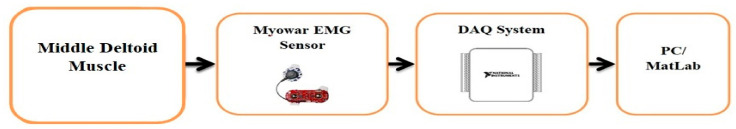
Experiment Setup and Configuration.

The analogue signal that was obtained was then digitized using the Data Acquisition system (USB6001 DAQ system) produced by National Instruments. The digitized signal was sent to a PC for further analysis. The USB6001 DAQ system is characterized by a resolution of 14 bits and sampling frequency of 20,000 samples/s. However, the acquired analogue signal was sampled at a frequency of 2000 Hz, which ultimately prevented Nyquist aliasing and reduced the computation time. The experimental setup and configuration are shown in Figure 2.

### 2.5. Pre-Processing of sEMG Signal

The digitized sEMG signal was recorded using an analogue input recorder application provided by Matlab (2018). The recorded sEMG signal was processed and filtered as follows:

I. The DC offset was first eliminated. Matlab provides an efficient function for removing the DC level that the raw EMG data is mounted on [52].
II. A nonzero-lag second order Butterworth IIR band-pass filter with a cut-off frequency of 25–350 Hz was applied to extract the beneficial EMG frequencies [20,31,44]. A Butterworth filter was selected as it provides a maximum flat response among the cut-off frequencies [52].
III. A nonzero-lag second order Butterworth IIR band-stop filter, with a cut-off frequency of 47–53 Hz, was also applied to eliminate the 50 Hz power line frequency.

**Figure 3:**
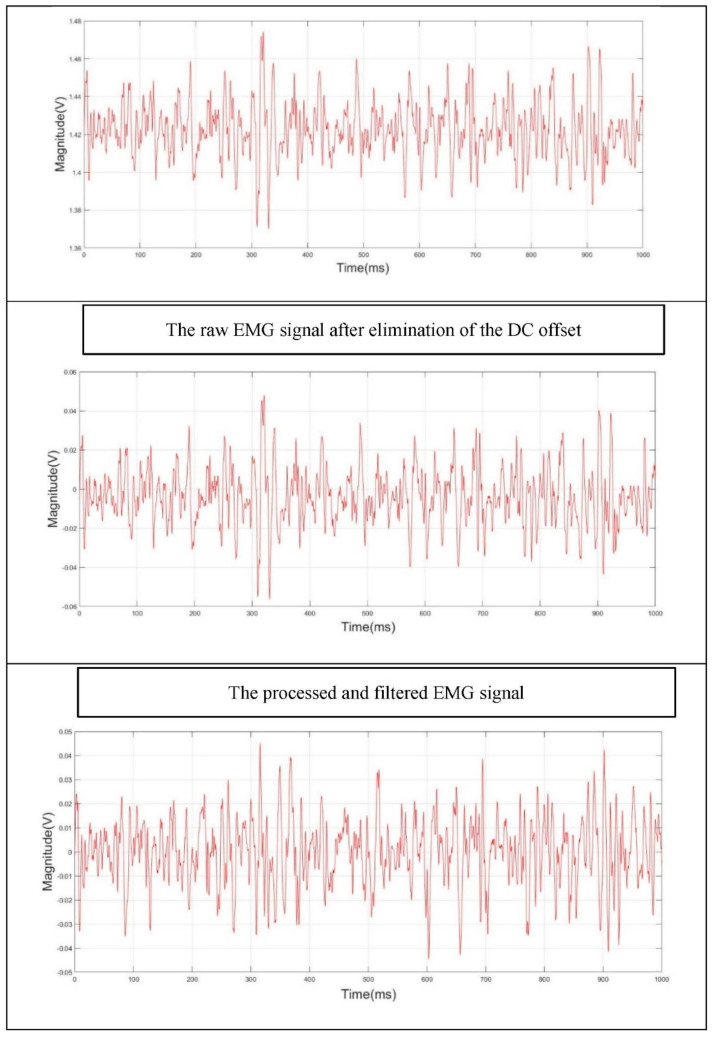
Steps for the processing and filtering of the raw EMG signal.

Figure 3 shows the steps for the processing and filtering of the raw EMG signal.

Next, a proper segmentation was applied to the processed and filtered EMG signal prior to the application of the proposed algorithm. The segmentation process was an important step to show how the fatigue progressed with time. A rectangular window of 3 s and an overlap of 1.5 s were used to segment the recorded raw EMG data. In other words, the entire EMG signal that was recorded was segmented into 3 s windows, after which the proposed algorithm was applied to these segments. Such a segmentation was chosen to provide real-time monitoring of muscle fatigue [53].

### 2.6. Double-Step Binary Classifier and Fatigue Index

The proposed algorithm involved analyzing the acquired sEMG signal in the time and frequency domains, respectively. The first step in analyzing the sEMG in the time domain using the proposed classifier involved the extraction of a time-domain feature, specifically the integrated EMG (IEMG). The IEMG is characterized by a low computation time, and can be represented as follows:

(2) $$IEMGi=\sum_{n=0}^{N-1}|EMG_{n}\;|$$

where i denotes the number of segments, and N represents the length of the segment.

It was previously proven that the IEMG gradually increases with the progress of muscle fatigue [54,55]. Therefore, the IEMG value was used to detect the progress of fatigue by comparing its current value with its initial value. The initial value of the IEMG (IEMGinitial) was the IEMG value of the first segment, whereas the current value of the IEMG (IEMGcurrent) referred to the IEMG value of the subsequent segments. Once the IEMGcurrent exceeded the IEMGinitial, the second step of the classifier was performed.

The second step involved analyzing the acquired sEMG signal in the frequency domain to differentiate between the high-frequency and low-frequency components of the EMG signal. Basically, the segmented EMG signal was filtered by two band-pass filters separately to produce two sub-signals, namely, a high-frequency sub-signal (HFSS) and a low-frequency sub-signal (LFSS). Then, the instantaneous mean amplitude (IMA) was calculated for the two sub-signals to ultimately obtain the fatigue index, which represents the difference between the IMA values of the LFSS and HFSS, respectively.

The boundaries of the high-frequency components (HFC) and low-frequency components (LFC) of the EMG had to be identified first for the accurate extraction of the HFSS and LFSS. The high-frequency components of the EMG had been previously identified to be in the range of 80–350 Hz [29]. Consequently, the LFC lay in the range of 25–79 Hz, which was also in agreement with the literature [13]. Furthermore, the mean frequency (MNF) and median frequency (MDF) were calculated to ensure an accurate identification of the frequency bands of the EMG. The MNF and MDF were chosen for their property in identifying the central frequency [16,56]. For instance, the MNF is the sum of the product of the EMG power spectrum and the frequency divided by the total sum of the power spectrum. In addition, the MDF is the frequency that divides the spectrum of the EMG into two regions with equal amplitudes [16]. The MNF and MDF are mathematically represented as follows:

(3) $$MNF_{i}=\overset{n}{\underset{k=1}{\sum }}f_{k}P_{k}\;/\;\overset{n}{\underset{k=1}{\sum }}P_{k}$$

(4) $$\sum_{k=1}^{m}P_{k}=\sum_{m}^{n}P_{k}=\frac{1}{2}\sum_{k=1}^{n}P_{k}$$

(5) $$MDF_{i}=fm$$

where n denotes the length of the segment, $P_{k}$ denotes the power spectrum value at bin, k, *m* denotes the frequency associated with the median frequency (*fm*), and i the number of segments [16,57].

Therefore, the mean frequency (MNF) and the median frequency (MDF) of the first EMG segment in fatigue condition for all the subjects were calculated (Table 3). This shows that MNF and MDF values mostly fell within the 75–85 Hz range, which confirmed that 80 Hz could be used as the frequency of separation between the HFC and LFC.

Once the frequency ranges for the LFC and HFC had been identified, each segment of the segmented EMG was filtered by two nonzero-lag fourth-order Butterworth band-pass filters. The first band-pass filter had a cut-off frequency of 25–79 Hz, and was used to extract the *LFSS*, while the second band-pass filter had a cut-off frequency of 80–350 Hz, and was used to extract the *HFSS*.

At this stage, each segment was filtered, and the *LFSS* and *HFSS* were successfully extracted. Then, the fast Fourier transform (FFT) was applied to these sub-signals to produce the *LFSSf* and *HFSSf*, respectively [58].

On applying the FFT to the *LFSS* and *HFSS*, which had been previously extracted from the original acquired EMG, a complex point was produced. Each complex point embraced real and imaginary parts. Then, the proposed algorithm calculated the instantaneous mean amplitude (*IMA*), as shown in Equations (6) and (7).

(6) $$IMA_{LFSSfi}=\frac{\;\sum_{n=0}^{N-1}|LFSSf_{i\left[n\right]}\;|\;}{N}$$

(7) $$IMA_{HFSSfi}=\frac{\;\sum_{n=0}^{N-1}|HFSSf_{i\left[n\right]}\;|\;}{N}$$

Eventually, the fatigue index, representing the *IMA* of the *LFSS* minus the *IMA* of the *HFSS*, was calculated using Equation (8) below:

(8) $$Fatigue\_Index=IMA_{LFSSfi}-IMA_{HFSSfi}$$

where N denotes the length of the segment, and i denotes the current segment.

Then, a decision was made based on the values of the Fatigue_index as follows:

(9) $$Fatigue\_Index=\left\{\begin{array}{c} \geq 0\;fatigue\;level\;reached\;\\ <0\;Otherwise\; \end{array}\right.$$

**Figure 4:**
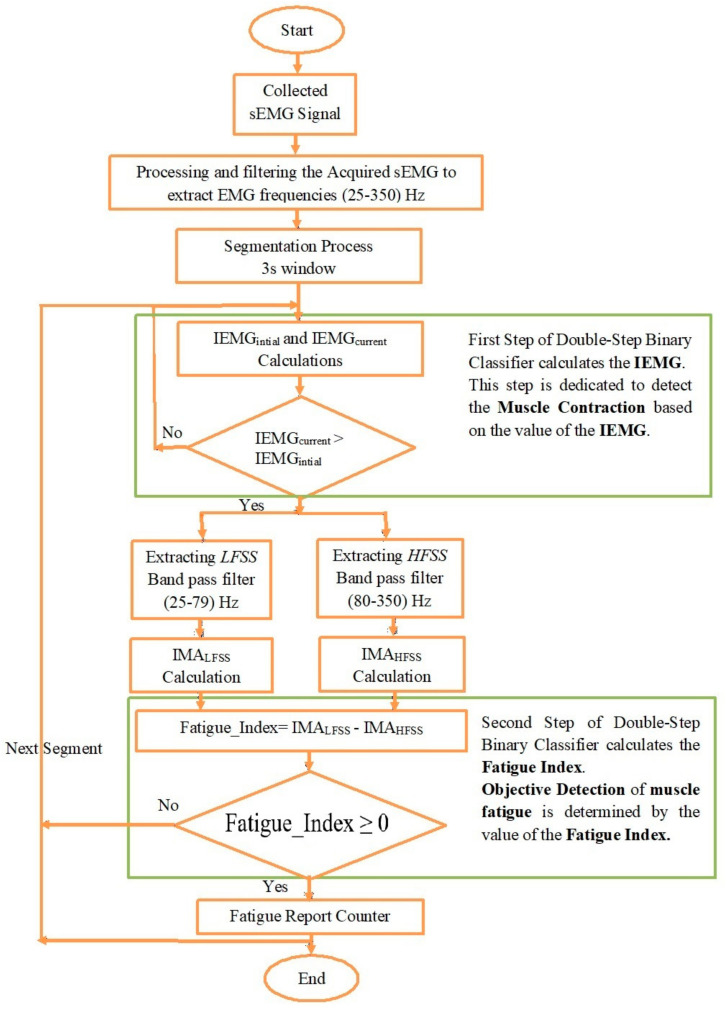
Flow chart of the proposed algorithm.

Equation (8) shows that the objective detection of muscle fatigue was determined by the value of the fatigue index. Figure 4 summaries the steps of the proposed algorithm. A fatigue report counter was added at the end of the algorithm to record the fatigue indices of the analyzed EMG segments, and to assign them as fatigue or non-fatigue segments throughout the session, as shown in the next section.

The proposed algorithm indicated that the IMA of the LFSS started at its minimum value in the first segment and tended to increase with the progression of fatigue. In contrast, the *IMA* of the *HFSS* started at its maximum value in the first segment and tended to decrease with the progression of fatigue. Therefore, the fatigue index was transformed from a negative value to a positive value throughout the fatigue progression. Muscle fatigue was found to have occurred and was objectively detected when the value of the proposed fatigue index was equal to or greater than zero. In other words, fatigue was reached when the *IMA* of the *LFSS* was equal to or greater than the *IMA* of the *HFSS*. Furthermore, the increase in the fatigue index was a clear indication of progressive fatigue. Moreover, if there was no change in the fatigue index, this would indicate that the muscle was relatively not experiencing progressive fatigue.

Consequently, the accuracy of the proposed algorithm was evaluated based on its ability to produce a zero or positive fatigue index once the subjects reported fatigue. As the sEMG signal was segmented prior to the calculation of the fatigue index, the algorithm was therefore said to be accurate if it was able to produce a zero or positive fatigue index in the last segment, when the individual reported fatigue. Furthermore, the first segments should produce a negative fatigue index and be assigned as non-fatigue. Thus, the analyzed data that were extracted from the fatigue condition were used to evaluate the accuracy of the algorithm, and this will be explained in the next section.

## 3. Results and Discussion

**Figure 5:**
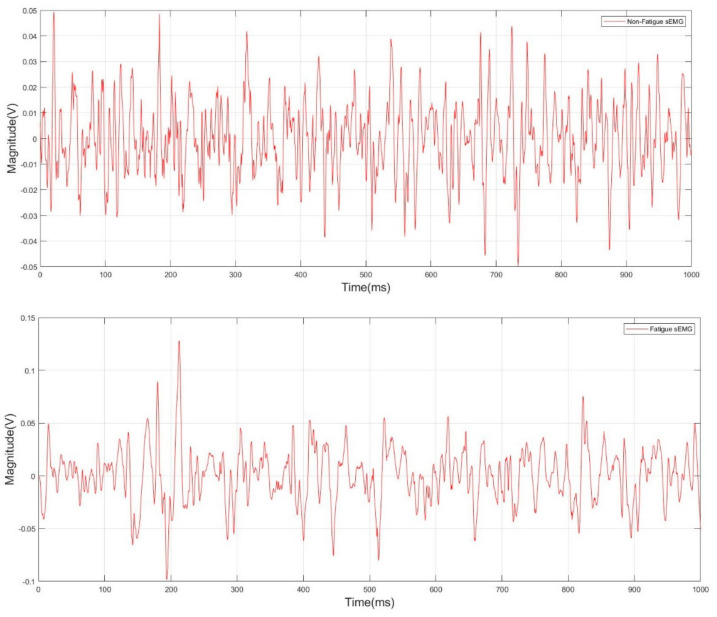
Non-fatigue and fatigue filtered sEMG signal.

A representation of the non-fatigued and fatigued filtered sEMG signal is shown in Figure 5. The variations between the non-fatigued and fatigued sEMG signals depend on the physiological parameters of the muscle, including the motor unit recruitment, fiber firing rate, fiber type, and the conduction velocity. The amplitude and frequency of the acquired sEMG signal were the main EMG characteristics that were affected by the aforementioned parameters [26].

As the proposed algorithm involved the extraction of time and frequency features from the acquired sEMG signal, the analyses in the time and frequency domains were therefore shown, respectively. First, the double-step binary classifier calculated the value of the integrated EMG (IEMG) to detect the initial contraction of the investigated muscle. The linear regression technique was used to identify the slope behavior of the IEMG throughout the progression of fatigue. Table 4 shows the slope values of the IEMG for the non-fatigue and fatigue conditions of all the subjects. It was clearly shown that the IEMG exhibited a positive slope in muscle fatigue condition.

**Figure 6:**
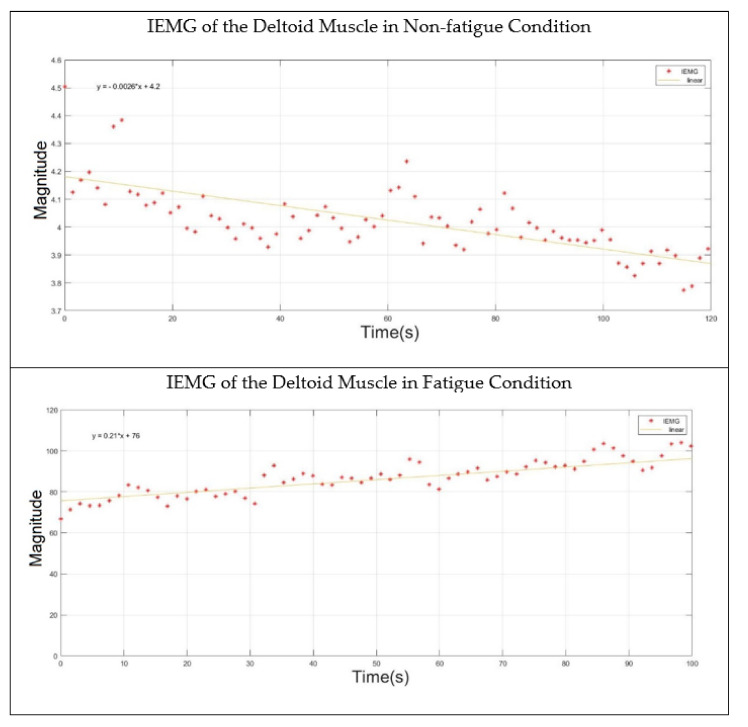
IEMG behavior of the deltoid muscle in non-fatigue and fatigue states.

The paired two-tail *t*-test was applied to identify the statistical difference between the IEMG slopes for both the fatigue and non-fatigue conditions of all subjects. At a confidence interval of 95%, the null hypothesis was rejected if the *p*-value was less than 0.05. Hence, the *p*-value was calculated and found to be 0.035, which indicated that there was a statistical difference between the two conditions in terms of the IEMG values. Furthermore, Figure 6 shows the behavior of the IEMG value while the deltoid muscle was in the non-fatigue and fatigue condition.

**Figure 7:**
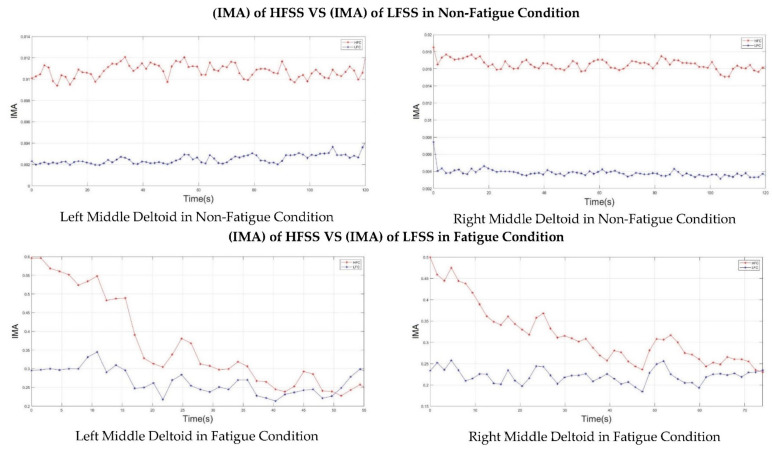
The IMA of the HFSS (red line) and LFSS (blue Line) of the middle deltoid muscle in non-fatigue condition (**top**) and in fatigue condition (**bottom**).

Second, the double-step binary classifier calculates the fatigue index based on the values of the instantaneous mean amplitude (IMA) of high-frequency and low-frequency sub-signals (HFSS and LFSS). Therefore, it was better to show how the IMA of the HFSS and LFSS behaved during the two muscle fatigue conditions (non-fatigue and fatigue). Figure 7 shows the IMA of both the HFSS and LFSS when the middle deltoid was in non-fatigue and fatigue conditions. In the non-fatigue condition, it was clearly shown that the IMA values of both the HFSS and LFSS were totally separated and the fatigue index exhibited a negative value. Hence, the fatigue did not progress. As mentioned in the previous section, even though the subjects performed no action while in the non-fatigue state, the middle deltoid muscle still played an important role in the stabilization of the shoulder joint, and ultimately produced a sort of sEMG signal.

When in a fatigue state, Figure 7 shows that the magnitude of the IMA for both the HFSS and LFSS started to change and intersected at some point, which indicated the presence of fatigue. In other words, the fatigue index was transformed from a negative to a positive value because of the rapid decrease in the IMA of the HFSS and an increase in the IMA of the LFSS. The transformation in the fatigue index indicated the objective detection of muscle fatigue.

Furthermore, Figure 7 shows that the right middle deltoid required a longer time than the left middle deltoid to experience fatigue for the same subject. Despite the fact that the left deltoid muscle experienced fatigue at 50 s, the IMA values of both the HFSS and LFSS continued to change, where the IMA of the HFSS continued to decrease while the IMA of the LFSS continued to increase after the onset of fatigue. This was attributed to the ability of the subject to resist fatigue for a further period after the onset of fatigue.

Figure 7 shows that the scapular plane of the upper limb accelerated the progress of muscle fatigue. Moreover, adding weights to the forearm substantially affected the IMA of both the HFSS and LFSS.

In terms of numbers, the fatigue indices in fatigue condition for each subject in the first and last segments (when their muscles had reached the fatigue level) are shown in Table 5.

**Figure 8:**
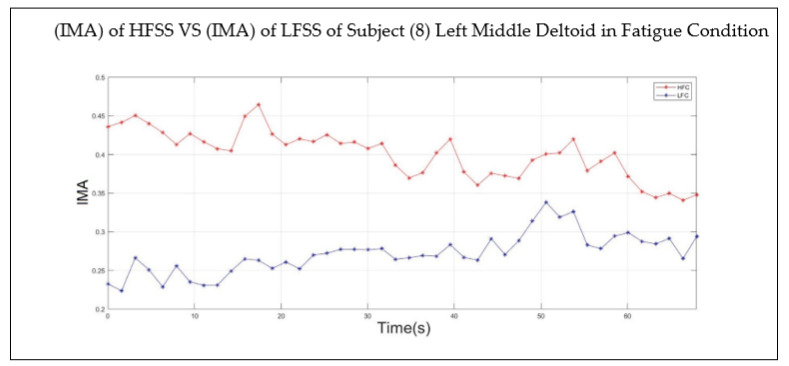
Behavior of IMA of the HFSS (red line) and LFSS (blue line) of the left middle deltoid muscle of subject 8 in fatigue condition.

Table 5 emphasizes that the fatigue index of all the subjects started with a negative value at the beginning of the session and was transformed to a positive value at the end of the session, when the fatigue level was reached. Although subjects 8, 12, 16, 22, and 32 reported the presence of fatigue in their middle deltoid muscles at the end of the session, their fatigue index was not transformed to a positive value. Thus, the behavior of the IMA of both the LFSS and HFSS for the aforementioned subjects was investigated. Figure 8 shows that the IMA values of both the HFSS and LFSS of subject 8 started to change, but had yet to intersect. In other words, the fatigue index started with a negative value and continued to increase throughout the session; however, it was not transformed to zero or to a positive value.

Table 5 also shows that the participants exhibited different timelines prior to the onset of fatigue. In other words, the participants varied in their ability to withstand fatigue throughout the session. Consequently, the proposed algorithm was able to objectively detect the fatigue despite individual variations.

For the purpose of an accurate evaluation and in considering each sEMG signal as having segments of length N, the fatigue indices of the first and last three sEMG segments of all the subjects in fatigue condition were calculated. A fatigue index with a negative value was an indication of non-fatigue, while a zero or positive value was an indication of fatigue, as shown in Table 6.

Table 6 shows that the first segments produced a negative fatigue index, thereby indicating that no fatigue had occurred yet. However, most of the last segments produced a positive fatigue index, and ultimately fatigue was reached. The results of the last two segments, either fatigue or non-fatigue, were considered for the mathematical calculation of the sensitivity, specificity, positive predictive value, and accuracy of the algorithm. The non-fatigue state at the (N − 1) segment represents a true negative (TN) classification of the proposed algorithm, while the fatigue state at the (N) segment represents a true positive (TP) classification. Consequently, the fatigue and the non-fatigue states in the (N − 1) and (N) segments represent the false positive (FP) and false negative, respectively. As shown in Table 6, the last two segment embrace a total of 70 TP, 72 TN, 3 FP, and 5 FN. The following are the mathematical representations of the aforementioned characteristics:

(10) $$Sensitivity=\frac{\;\mathrm{TP}\;}{\mathrm{TP}+\mathrm{FN}}=0.9333\;\approx \;93\%$$

(11) $$Specificty=\frac{\;\mathrm{TN}\;}{\mathrm{TN}+\mathrm{FP}}=0.96=96\%$$

(12) $$Positive\;Predictive\;Value=\frac{\;\mathrm{TP}\;}{\mathrm{TP}+\mathrm{FP}}=0.9589\;\approx \;96\%$$

(13) $$Accuracy=\frac{\;\mathrm{TN}+\mathrm{TP}\;}{\mathrm{TN}+\mathrm{TP}+\mathrm{FN}+\mathrm{FP}}=0.9466\;\approx \;95\%$$

As mentioned in the earlier section on the literature review, various classifiers were used in an attempt to objectively detect fatigue. Thus, Table 7 shows a comparison between the proposed algorithm and other algorithms to emphasize the differences in terms of the employed classifier, and the accuracy obtained.

As shown in Table 7, the proposed algorithm showed the highest accuracy in comparison to previous studies. It should be noted that non-adult individuals were excluded from the study as the skeletal muscles of adults and non-adults may respond differently [62]. For instance, the small-sized muscles of non-adults could result in less accurate EMG signals. Moreover, the ability of non-adults to withstand the fatigue in comparison to adults [62].

The calculation of the IEMG value as the first step in the proposed algorithm was considered as an important procedure to accurately identify the fatigue condition. The importance of this step came from its ability to distinguish between relaxed and contracted muscles. Relaxed muscles exhibit no significant EMG and have a different power spectrum than contracted muscles [11,63]. This could ultimately affect the second step (fatigue index calculation) of the proposed classifier. More specifically, the first step of the proposed algorithm was fully aimed at ensuring that only the EMG signals that were extracted from the contracted muscles were analyzed by the second step of the proposed classifier. Applying only the second step on signal acquired from relaxed muscle could produce false positive fatigue index. In this study, however, the middle deltoid muscle was investigated, which exhibits a sort of contraction in both non-fatigue and fatigue conditions that helps in stabilizing the shoulder joint [40].

**Figure 9:**
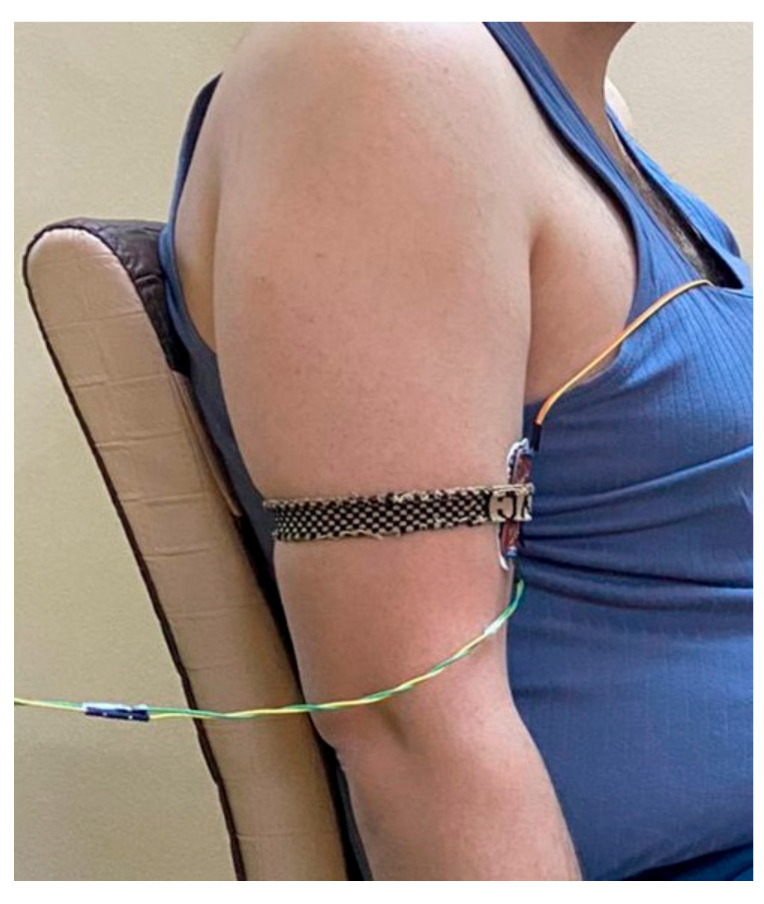
Acquiring the signal from the bicep muscle at relaxed state.

**Figure 10:**
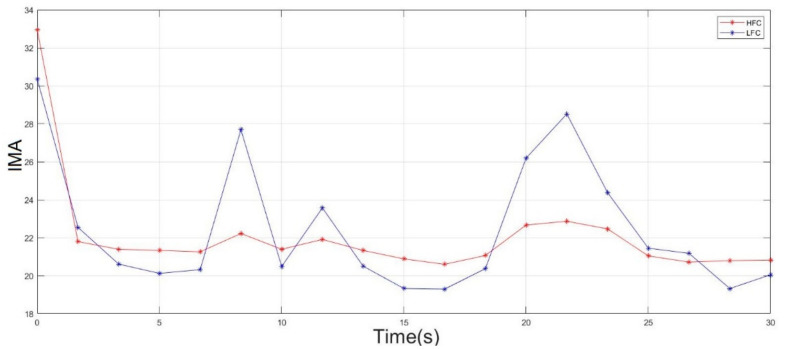
Behavior of IMA of the HFSS (red line) and LFSS (blue line) of the biceps at relaxed state.

Consequently, the second step of the proposed algorithm was applied on a relaxed muscle’s signal to practically approve the necessity of the first step. Ref. [64] have clarified that biceps are virtually silent and eventually can be considered relaxed when the elbow is extended. Hence, the bicep signals at the relaxed state of eight subjects (Figure 9) were acquired and analyzed only by the second step of the proposed algorithm. Figure 10 shows how the IMA values of HFSS and LFSS fluctuate and intersect, which ultimately produces a false positive fatigue index throughout the session.

Several factors were precisely considered to ensure that the results obtained were reliable. First, the weight was clutched on the forearm instead of being held by the hand to avoid fatigue in the hand muscles prior to fatigue in the deltoid muscles. Holding the weight by the hand would incline the subject to report an uncomfortable feeling caused by fatigue in the hand muscles.

Second, special attention was also given to the Myoware muscle sensor to ensure that the sEMG signal acquired would be accurate. For instance, the two main electrodes of the sensor were placed on the belly of the muscle and in a straight position, while the reference electrode was placed on the bony or different tissue [47,48]. The wrong attachment of the sensor could substantially affect the accuracy and strength of the acquired signal.

Third, it was reported that the loose attachment between the sensor and the muscle could dramatically affect the acquired signal because of any sudden movement of the loosened sensor [46]. Therefore, a tie was applied to prevent the sensor from being loosely attached to the muscle.

Finally, the sampling frequency was another issue that had to be accurately identified prior to the processing of the acquired signal [65]. The highest frequency of the EMG was identified and found to be 400 Hz. Hence, the minimum sampling frequency had to be no less than 800 Hz to prevent Nyquist aliasing. Despite the fact that increasing the sampling frequency above the Nyquist rate could provide more information about the acquired signal, a higher sampling frequency would compromise the real-time processing [66].

## 4. Conclusions

An algorithm employing a new fatigue index was proposed for the objective detection of muscle fatigue. The proposed algorithm analyzed the acquired sEMG signal in the time and frequency domains to extract the IEMG and IMA features, respectively to ultimately identify the fatigue index. It was found that a gradual increase in the IEMG value was a clear indication of muscle contraction. Furthermore, the instantaneous mean amplitude (IMA) of the high-frequency sub-signal (HFSS) and low-frequency sub-signal followed different behaviors. The IMA of the HFSS tended to decrease during the progression of fatigue, while the IMA of the LFSS tended to increase. Thus, the fatigue index was identified as the difference between the IMA values of the LFSS and HFSS, respectively. Muscle fatigue was found to be present and was objectively detected when the value of the proposed fatigue index was equal to or greater than zero.

## Figures and Tables

**Table 1:** Mean accuracy of the reviewed preliminary EMG studies.

Ref	Accuracy %
[34]	88.4
[35]	90
[36]	90
[37]	93.5
[38]	94
[39]	79.4
	Mean (***μ***) = 89.21

**Table 2:** Parameters of the Myoware muscle sensor.

Parameter	Detail
Input Impedance	110 GΩ
Supply Voltage	3.3 V
Common Mode Rejection Ratio (CMRR)	110
Gain	Adjustable

**Table 3:** MNF and MDF values for deltoid muscle in fatigue condition.

Reference	Middle Deltoid Muscle	Mean Frequency (MNF) Hz	Median Frequency (MDF) Hz	Reference	Middle Deltoid Muscle	Mean Frequency (MNF) Hz	Median Frequency (MDF) Hz
Subject 1	Left muscle	85	73	Subject 20	Left muscle	79	72
	Right muscle	84	76		Right muscle	85	76
Subject 2	Left muscle	83	77	Subject 21	Left muscle	78	70
	Right muscle	98	90		Right muscle	84	78
Subject 3	Left muscle	88	80	Subject 22	Left muscle	84	76
	Right muscle	Not-Examined	Not-Examined		Right muscle	86	78
Subject 4	Left muscle	85	70	Subject 23	Left muscle	83	75
	Right muscle	84	73		Right muscle	79	74
Subject 5	Left muscle	88	79	Subject 24	Left muscle	87	79
	Right muscle	89	80		Right muscle	72	68
Subject 6	Left muscle	77	72	Subject 25	Left muscle	75	70
	Right muscle	89	84		Right muscle	85	75
Subject 7	Left muscle	69	67	Subject 26	Left muscle	86	80
	Right muscle	65	63		Right muscle	83	75
Subject 8	Left muscle	89	80	Subject 27	Left muscle	93	86
	Right muscle	83	77		Right muscle	83	75
Subject 9	Left muscle	86	73	Subject 28	Left muscle	89	77
	Right muscle	96	89		Right muscle	83	76
Subject 10	Left muscle	89	81	Subject 29	Left muscle	77	73
	Right muscle	90	83		Right muscle	86	78
Subject 11	Left muscle	86	75	Subject 30	Left muscle	85	80
	Right muscle	92	85		Right muscle	90	80
Subject 12	Left muscle	87	80	Subject 31	Left muscle	89	82
	Right muscle	89	82		Right muscle	84	75
Subject 13	Left muscle	88	78	Subject 32	Left muscle	82	74
	Right muscle	93	84		Right muscle	84	76
Subject 14	Left muscle	83	74	Subject 33	Left muscle	81	75
	Right muscle	81	73		Right muscle	85	74
Subject 15	Left muscle	82	74	Subject 34	Left muscle	86	74
	Right muscle	84	79		Right muscle	83	76
Subject 16	Left muscle	86	75	Subject 35	Left muscle	85	78
	Right muscle	85	76		Right muscle	83	76
Subject 17	Left muscle	82	74	Subject 36	Left muscle	93	88
	Right muscle	87	77		Right muscle	79	73
Subject 18	Left muscle	81	74	Subject 37	Left muscle	89	82
	Right muscle	92	86		Right muscle	80	72
Subject 19	Left muscle	80	72	Subject 38	Left muscle	87	76
	Right muscle	87	81		Right muscle	84	76

**Table 4:** Slopes of IEMG for fatigue and non-fatigue conditions.

Reference	Middle Deltoid Muscle	Slope’s Value Non-Fatigue	Slope’s Value Fatigue	Reference	Middle Deltoid Muscle	Slope’s Value Non-Fatigue	Slope’s Value Fatigue
Subject 1	Left muscle	−0.0018	0.038	Subject 20	Left muscle	−0.00011	0.037
	Right muscle	−0.0038	0.4		Right muscle	−0.00004	0.045
Subject 2	Left muscle	−0.00016	0.14	Subject 21	Left muscle	−0.0002	0.203
	Right muscle	−0.0002	1.4		Right muscle	−0.00234	0.038
Subject 3	Left muscle	−0.00081	0.15	Subject 22	Left muscle	−0.0018	0.04
	Right muscle	Not-Examined	Not-Examined		Right muscle	−0.0007	0.8
Subject 4	Left muscle	−0.0021	0.057	Subject 23	Left muscle	−0.00016	0.16
	Right muscle	−0.0016	0.074		Right muscle	−0.0002	1.8
Subject 5	Left muscle	−0.00021	0.0014	Subject 24	Left muscle	−0.00009	0.6
	Right muscle	−0.00001	0.014		Right muscle	−0.0021	0.1
Subject 6	Left muscle	−0.00015	0.33	Subject 25	Left muscle	0.0008	0.08
	Right muscle	−0.00008	0.28		Right muscle	−0.00021	0.002
Subject 7	Left muscle	−0.0017	0.15	Subject 26	Left muscle	−0.0056	0.023
	Right muscle	−0.0014	0.098		Right muscle	−0.00078	0.23
Subject 8	Left muscle	−0.0046	0.048	Subject 27	Left muscle	−0.0067	0.28
	Right muscle	−0.0043	0.053		Right muscle	−0.0067	0.18
Subject 9	Left muscle	−0.0013	0.27	Subject 28	Left muscle	−0.009	0.1
	Right muscle	−0.00008	0.09		Right muscle	−0.0067	0.05
Subject 10	Left muscle	−0.005	0.087	Subject 29	Left muscle	−0.0021	0.06
	Right muscle	−0.0073	0.09		Right muscle	0.0034	0.15
Subject 11	Left muscle	−0.0002	0.024	Subject 30	Left muscle	−0.00076	0.1
	Right muscle	−0.00023	0.059		Right muscle	−0.0099	0.02
Subject 12	Left muscle	−0.0044	0.027	Subject 31	Left muscle	−0.00045	0.96
	Right muscle	−0.000013	0.0044		Right muscle	−0.0002	0.024
Subject 13	Left muscle	−0.00067	0.036	Subject 32	Left muscle	−0.0045	0.033
	Right muscle	−0.0026	0.21		Right muscle	−0.0045	0.03
Subject 14	Left muscle	−0.00001	0.039	Subject 33	Left muscle	−0.344	0.005
	Right muscle	−0.000005	0.022		Right muscle	−0.00067	0.068
Subject 15	Left muscle	−0.000007	0.045	Subject 34	Left muscle	−0.00045	0.2
	Right muscle	−0.00057	0.024		Right muscle	−0.00001	0.04
Subject 16	Left muscle	−0.00023	0.02	Subject 35	Left muscle	−0.004	0.05
	Right muscle	−0.00012	0.019		Right muscle	−0.0067	0.05
Subject 17	Left muscle	−0.0009	0.047	Subject 36	Left muscle	−0.00057	0.033
	Right muscle	−0.0022	0.019		Right muscle	−0.00023	0.02
Subject 18	Left muscle	−0.0033	0.085	Subject 37	Left muscle	−0.0067	0.03
	Right muscle	−0.00067	0.011		Right muscle	−0.0006	0.06
Subject 19	Left muscle	−0.0006	0.05	Subject 38	Left muscle	−0.000046	0.035
	Right muscle	−0.0003	0.05		Right muscle	−0.0000078	0.019

**Table 5:** Fatigue indices in fatigue condition.

Reference	Middle Deltoid Muscle	Fatigue Index Value		Time Consumed (s)	Reference	Middle Deltoid Muscle	Fatigue Index Value		Time Consumed (s)
		First Segment (No Fatigue)	Last Segment (Fatigue Reached)				First Segment (No Fatigue)	Last Segment (Fatigue Reached)	
Subject 1	Left muscle	−0.682	0.063	25	Subject 20	Left muscle	−0.438	0.068	41
	Right muscle	−0.648	0.072	31		Right muscle	−0.398	0.045	56
Subject 2	Left muscle	−0.468	0.006	33	Subject 21	Left muscle	−0.425	0.041	44
	Right muscle	−0.248	0.003	60		Right muscle	−0.438	0.009	68
Subject 3	Left muscle	−0.314	0.010	70	Subject 22	Left muscle	−0.145	0.019	94
	Right muscle	Not-Examined	Not-Examined	Not-Examined		Right muscle	−0.245	0.067	100
Subject 4	Left muscle	−0.122	0.021	94	Subject 23	Left muscle	−0.488	0.017	67
	Right muscle	−0.142	0.096	100		Right muscle	−0.134	0.003	83
Subject 5	Left muscle	−0.300	0.020	51	Subject 24	Left muscle	−0.257	0.001	85
	Right muscle	−0.266	0.004	113		Right muscle	−0.490	0.011	95
Subject 6	Left muscle	−0.239	0.002	109	Subject 25	Left muscle	−0.130	0.023	53
	Right muscle	−0.558	0.001	115		Right muscle	−0.290	−0.023	34
Subject 7	Left muscle	−0.126	0.033	67	Subject 26	Left muscle	−0.290	0.027	42
	Right muscle	−0.253	0.050	75		Right muscle	−0.309	0.012	54
Subject 8	Left muscle	−0.203	−0.053	12	Subject 27	Left muscle	−0.463	0.018	80
	Right muscle	−0.326	0.022	20		Right muscle	−0.523	0.017	85
Subject 9	Left muscle	−0.363	0.027	85	Subject 28	Left muscle	−0.285	0.008	49
	Right muscle	−0.450	0.012	92		Right muscle	−0.137	0.062	76
Subject 10	Left muscle	−0.431	0.005	45	Subject 29	Left muscle	−0.389	0.010	70
	Right muscle	−0.428	0.071	83		Right muscle	−0.652	0.023	76
Subject 11	Left muscle	−0.358	0.020	73	Subject 30	Left muscle	−0.398	0.023	81
	Right muscle	−0.454	0.003	75		Right muscle	−0.378	0.034	89
Subject 12	Left muscle	−0.424	−0.012	65	Subject 31	Left muscle	−0.289	0.028	59
	Right muscle	−0.473	0.023	66		Right muscle	−0.478	0.035	69
Subject 13	Left muscle	−0.479	0.037	61	Subject 32	Left muscle	−0.145	0.012	65
	Right muscle	−0.518	0.012	74		Right muscle	−0.537	−0.059	59
Subject 14	Left muscle	−0.396	0.019	56	Subject 33	Left muscle	−0.405	0.047	60
	Right muscle	−0.439	0.033	67		Right muscle	−0.407	0.063	72
Subject 15	Left muscle	−0.427	0.009	58	Subject 34	Left muscle	−0.537	0.059	59
	Right muscle	−0.363	0.045	64		Right muscle	−0.469	0.033	85
Subject 16	Left muscle	−0.415	−0.079	52	Subject 35	Left muscle	−0.425	0.006	63
	Right muscle	−0.340	0.023	92		Right muscle	−0.526	0.032	78
Subject 17	Left muscle	−0.352	0.016	52	Subject 36	Left muscle	−0.397	0.048	49
	Right muscle	−0.523	0.002	78		Right muscle	−0.465	0.063	65
Subject 18	Left muscle	−0.392	0.098	52	Subject 37	Left muscle	−0.324	0.055	59
	Right muscle	−0.560	0.003	69		Right muscle	−0.372	0.034	73
Subject 19	Left muscle	−0.521	0.004	49	Subject 38	Left muscle	−0.426	0.056	64
	Right muscle	−0.482	0.022	65		Right muscle	−0.293	0.036	70

**Table 6:** Accuracy of the proposed algorithm.

Reference	Middle Deltoid Muscle	Segments Fatigue Classification					
		1st Seg.	2nd Seg.	3rd Seg.	(N−2)th Seg.	(N−1)th Seg.	Nth Seg.
**Subject 1**	Left muscle	Non-fatigue	Non-fatigue	Non-fatigue	Non-fatigue	Non-fatigue	Fatigue
	Right muscle	Non-fatigue	Non-fatigue	Non-fatigue	Non-fatigue	Non-fatigue	Fatigue
**Subject 2**	Left muscle	Non-fatigue	Non-fatigue	Non-fatigue	Non-fatigue	Non-fatigue	Fatigue
	Right muscle	Non-fatigue	Non-fatigue	Non-fatigue	Non-fatigue	Fatigue	Fatigue
**Subject 3**	Left muscle	Non-fatigue	Non-fatigue	Non-fatigue	Non-fatigue	Non-fatigue	Fatigue
	Right muscle	Not-Examined	Not-Examined	Not-Examined	Not-Examined	Not-Examined	Not-Examined
**Subject 4**	Left muscle	Non-fatigue	Non-fatigue	Non-fatigue	Non-fatigue	Non-fatigue	Fatigue
	Right muscle	Non-fatigue	Non-fatigue	Non-fatigue	Non-fatigue	Non-fatigue	Fatigue
**Subject 5**	Left muscle	Non-fatigue	Non-fatigue	Non-fatigue	Non-fatigue	Non-fatigue	Fatigue
	Right muscle	Non-fatigue	Non-fatigue	Non-fatigue	Non-fatigue	Non-fatigue	Fatigue
**Subject 6**	Left muscle	Non-fatigue	Non-fatigue	Non-fatigue	Non-fatigue	Non-fatigue	Fatigue
	Right muscle	Non-fatigue	Non-fatigue	Non-fatigue	Non-fatigue	Non-fatigue	Fatigue
**Subject 7**	Left muscle	Non-fatigue	Non-fatigue	Non-fatigue	Non-fatigue	Non-fatigue	Fatigue
	Right muscle	Non-fatigue	Non-fatigue	Non-fatigue	Non-fatigue	Non-fatigue	Fatigue
**Subject 8**	Left muscle	Non-fatigue	Non-fatigue	Non-fatigue	Non-fatigue	Non-fatigue	Non-fatigue
	Right muscle	Non-fatigue	Non-fatigue	Non-fatigue	Non-fatigue	Non-fatigue	Fatigue
**Subject 9**	Left muscle	Non-fatigue	Non-fatigue	Non-fatigue	Non-fatigue	Non-fatigue	Fatigue
	Right muscle	Non-fatigue	Non-fatigue	Non-fatigue	Non-fatigue	Non-fatigue	Fatigue
**Subject 10**	Left muscle	Non-fatigue	Non-fatigue	Non-fatigue	Non-fatigue	Non-fatigue	Fatigue
	Right muscle	Non-fatigue	Non-fatigue	Non-fatigue	Non-fatigue	Non-fatigue	Fatigue
**Subject 11**	Left muscle	Non-fatigue	Non-fatigue	Non-fatigue	Non-fatigue	Non-fatigue	Fatigue
	Right muscle	Non-fatigue	Non-fatigue	Non-fatigue	Non-fatigue	Non-fatigue	Fatigue
**Subject 12**	Left muscle	Non-fatigue	Non-fatigue	Non-fatigue	Non-fatigue	Non-fatigue	Non-fatigue
	Right muscle	Non-fatigue	Non-fatigue	Non-fatigue	Non-fatigue	Non-fatigue	Fatigue
**Subject 13**	Left muscle	Non-fatigue	Non-fatigue	Non-fatigue	Non-fatigue	Non-fatigue	Fatigue
	Right muscle	Non-fatigue	Non-fatigue	Non-fatigue	Non-fatigue	Non-fatigue	Fatigue
**Subject 14**	Left muscle	Non-fatigue	Non-fatigue	Non-fatigue	Non-fatigue	Non-fatigue	Fatigue
	Right muscle	Non-fatigue	Non-fatigue	Non-fatigue	Non-fatigue	Non-fatigue	Fatigue
**Subject 15**	Left muscle	Non-fatigue	Non-fatigue	Non-fatigue	Non-fatigue	Non-fatigue	Fatigue
	Right muscle	Non-fatigue	Non-fatigue	Non-fatigue	Non-fatigue	Non-fatigue	Fatigue
**Subject 16**	Left muscle	Non-fatigue	Non-fatigue	Non-fatigue	Non-fatigue	Non-fatigue	Non-fatigue
	Right muscle	Non-fatigue	Non-fatigue	Non-fatigue	Non-fatigue	Non-fatigue	Fatigue
**Subject 17**	Left muscle	Non-fatigue	Non-fatigue	Non-fatigue	Non-fatigue	Non-fatigue	Fatigue
	Right muscle	Non-fatigue	Non-fatigue	Non-fatigue	Non-fatigue	Non-fatigue	Fatigue
**Subject 18**	Left muscle	Non-fatigue	Non-fatigue	Non-fatigue	Non-fatigue	Non-fatigue	Fatigue
	Right muscle	Non-fatigue	Non-fatigue	Non-fatigue	Non-fatigue	Non-fatigue	Fatigue
**Subject 19**	Left muscle	Non-fatigue	Non-fatigue	Non-fatigue	Non-fatigue	Non-fatigue	Fatigue
	Right muscle	Non-fatigue	Non-fatigue	Non-fatigue	Non-fatigue	Non-fatigue	Fatigue
**Subject 20**	Left muscle	Non-fatigue	Non-fatigue	Non-fatigue	Non-fatigue	Non-fatigue	Fatigue
	Right muscle	Non-fatigue	Non-fatigue	Non-fatigue	Non-fatigue	Non-fatigue	Fatigue
**Subject 21**	Left muscle	Non-fatigue	Non-fatigue	Non-fatigue	Non-fatigue	Non-fatigue	Fatigue
	Right muscle	Non-fatigue	Non-fatigue	Non-fatigue	Non-fatigue	Non-fatigue	Fatigue
**Subject 22**	Left muscle	Non-fatigue	Non-fatigue	Non-fatigue	Non-fatigue	Non-fatigue	Fatigue
	Right muscle	Non-fatigue	Non-fatigue	Non-fatigue	Non-fatigue	Non-fatigue	Fatigue
**Subject 23**	Left muscle	Non-fatigue	Non-fatigue	Non-fatigue	Non-fatigue	Non-fatigue	Fatigue
	Right muscle	Non-fatigue	Non-fatigue	Non-fatigue	Non-fatigue	Non-fatigue	Fatigue
**Subject 24**	Left muscle	Non-fatigue	Non-fatigue	Non-fatigue	Non-fatigue	Non-fatigue	Fatigue
	Right muscle	Non-fatigue	Non-fatigue	Non-fatigue	Non-fatigue	Non-fatigue	Fatigue
**Subject 25**	Left muscle	Non-fatigue	Non-fatigue	Non-fatigue	Non-fatigue	Non-fatigue	Fatigue
	Right muscle	Non-fatigue	Non-fatigue	Non-fatigue	Non-fatigue	Non-fatigue	Non-fatigue
**Subject 26**	Left muscle	Non-fatigue	Non-fatigue	Non-fatigue	Non-fatigue	Non-fatigue	Fatigue
	Right muscle	Non-fatigue	Non-fatigue	Non-fatigue	Non-fatigue	Non-fatigue	Fatigue
**Subject 27**	Left muscle	Non-fatigue	Non-fatigue	Non-fatigue	Non-fatigue	Non-fatigue	Fatigue
	Right muscle	Non-fatigue	Non-fatigue	Non-fatigue	Non-fatigue	Non-fatigue	Fatigue
**Subject 28**	Left muscle	Non-fatigue	Non-fatigue	Non-fatigue	Non-fatigue	Non-fatigue	Fatigue
	Right muscle	Non-fatigue	Non-fatigue	Non-fatigue	Non-fatigue	Non-fatigue	Fatigue
**Subject 29**	Left muscle	Non-fatigue	Non-fatigue	Non-fatigue	Non-fatigue	Fatigue	Fatigue
	Right muscle	Non-fatigue	Non-fatigue	Non-fatigue	Non-fatigue	Non-fatigue	Fatigue
**Subject 30**	Left muscle	Non-fatigue	Non-fatigue	Non-fatigue	Non-fatigue	Non-fatigue	Fatigue
	Right muscle	Non-fatigue	Non-fatigue	Non-fatigue	Non-fatigue	Non-fatigue	Fatigue
**Subject 31**	Left muscle	Non-fatigue	Non-fatigue	Non-fatigue	Non-fatigue	Non-fatigue	Fatigue
	Right muscle	Non-fatigue	Non-fatigue	Non-fatigue	Non-fatigue	Non-fatigue	Fatigue
**Subject 32**	Left muscle	Non-fatigue	Non-fatigue	Non-fatigue	Non-fatigue	Non-fatigue	Fatigue
	Right muscle	Non-fatigue	Non-fatigue	Non-fatigue	Non-fatigue	Non-fatigue	Non-fatigue
**Subject 33**	Left muscle	Non-fatigue	Non-fatigue	Non-fatigue	Non-fatigue	Non-fatigue	Fatigue
	Right muscle	Non-fatigue	Non-fatigue	Non-fatigue	Non-fatigue	Non-fatigue	Fatigue
**Subject 34**	Left muscle	Non-fatigue	Non-fatigue	Non-fatigue	Non-fatigue	Non-fatigue	Fatigue
	Right muscle	Non-fatigue	Non-fatigue	Non-fatigue	Non-fatigue	Non-fatigue	Fatigue
**Subject 35**	Left muscle	Non-fatigue	Non-fatigue	Non-fatigue	Non-fatigue	Non-fatigue	Fatigue
	Right muscle	Non-fatigue	Non-fatigue	Non-fatigue	Non-fatigue	Non-fatigue	Fatigue
**Subject 36**	Left muscle	Non-fatigue	Non-fatigue	Non-fatigue	Non-fatigue	Non-fatigue	Fatigue
	Right muscle	Non-fatigue	Non-fatigue	Non-fatigue	Non-fatigue	Non-fatigue	Fatigue
**Subject 37**	Left muscle	Non-fatigue	Non-fatigue	Non-fatigue	Non-fatigue	Non-fatigue	Fatigue
	Right muscle	Non-fatigue	Non-fatigue	Non-fatigue	Non-fatigue	Fatigue	Fatigue
**Subject 38**	Left muscle	Non-fatigue	Non-fatigue	Non-fatigue	Non-fatigue	Non-fatigue	Fatigue
	Right muscle	Non-fatigue	Non-fatigue	Non-fatigue	Non-fatigue	Non-fatigue	Fatigue

**Table 7:** Comparison between the proposed algorithm and three available algorithms.

Reference	Type of Employed Classifier	Obtained Accuracy
[59]	Support Vector Machine (SVM)	85.5%
[60]	Multilayer Perceptron (MLP)	86%
[26]	Support Vector Machine (SVM)	91.39%
[61]	Linear Discriminant Analysis (LDA)	88.41%
Our work	Double-Step Binary Classifier	94.66%

## Data Availability

The data presented in this study are available on request from the corresponding author. The data are not publically available due to privacy concerns.
